# Gut microbiome features in pediatric food allergy: a scoping review

**DOI:** 10.3389/falgy.2024.1438252

**Published:** 2024-09-25

**Authors:** Margherita Farnetano, Laura Carucci, Serena Coppola, Franca Oglio, Antonio Masino, Marica Cozzolino, Rita Nocerino, Roberto Berni Canani

**Affiliations:** ^1^ Department of Translational Medical Science, University of Naples Federico II, Naples, Italy; ^2^ ImmunoNutritionLab at the CEINGE Advanced Biotechnologies Research Center, University of Naples Federico II, Naples, Italy; ^3^ Department of Biomedicine and Prevention, University of Rome “Tor Vergata”, Rome, Italy; ^4^ Task Force on Microbiome Studies, University of Naples Federico II, Naples, Italy; ^5^ European Laboratory for the Investigation of Food-Induced Diseases, University of Naples Federico II, Naples, Italy

**Keywords:** microbiota, short-chain fatty acids, probiotics, allergy, dysbiosis, immune tolerance, cow milk protein allergy, children

## Abstract

Increasing evidence suggests that alterations in the gut microbiome (GM) play a pivotal role in the pathogenesis of pediatric food allergy (FA). This scoping review analyzes the current evidence on GM features associated with pediatric FAs and highlights the importance of the GM as a potential target of intervention for preventing and treating this common condition in the pediatric age. Following the Preferred Reporting Items for Systematic Reviews and Meta-Analysis guidelines, we searched PubMed and Embase using the keywords (gut microbiome OR dysbiosis OR gut microbiota OR microbiome signatures) AND (food allergy OR IgE-mediated food allergy OR food protein-induced allergic proctocolitis OR food protein-induced enterocolitis OR non-IgE food allergy OR cow milk allergy OR hen egg allergy OR peanut allergy OR fish allergy OR shellfish allergy OR tree nut allergy OR soy allergy OR wheat allergy OR rice allergy OR food sensitization). We included 34 studies reporting alterations in the GM in children affected by FA compared with healthy controls. The GM in pediatric FAs is characterized by a higher abundance of harmful microorganisms (e.g., Enterobacteriaceae, *Clostridium sensu stricto*, *Ruminococcus gnavus*, and *Blautia* spp.) and lower abundance of beneficial bacteria (e.g., Bifidobacteriaceae, *Lactobacillaceae*, some *Bacteroides* species). Moreover, we provide an overview of the mechanisms of action elicited by these bacterial species in regulating immune tolerance and of the main environmental factors that can modulate the composition and function of the GM in early life. Altogether, these data improve our knowledge of the pathogenesis of FA and can open the way to innovative diagnostic, preventive, and therapeutic strategies for managing these conditions.

## 1 Introduction

Food allergy (FA) is one of the most common chronic conditions in the pediatric age, and its prevalence has increased in recent decades (1). Increased FA prevalence is paralleled with a worsening of the clinical picture, leading to a significant increase in hospital admissions for food-induced anaphylaxis (2, 3). This dramatic scenario has a direct impact on the psychological burden of patients and their parents as well as on the costs for the families and healthcare systems (4, 5).

Food allergy results from a breakdown of the immune tolerance mechanisms (6). The induction and maintenance of immune tolerance is ensured by a dynamic and bidirectional link between the gut microbiome (GM) and immune system, defining the concept of the GM-immune system axis. Several environmental factors have been indicated as modulators of this axis, especially in the first 1,000 days of life (Table 1) (7–9). During this critical period, the development of the structure and function of the GM drives the development of the immune system (10). Thus, environmental factors, shaping the composition of the GM, influence immune system development and establish a child's current and future health status. Recent evidence shows that early exposure to drugs, such as antibiotics and gastric acidity inhibitors, antiseptic agents, ultra-processed foods (UPFs), and pollutants negatively influences the GM and increases the risk of developing chronic non-communicable diseases, including FA (10).

**Table 1:** Environmental factors that shape the composition of the gut microbiome of the child.

Gut microbiome alterations								
Environmental factors		Actinomycetota	Bacteroidota	Bacillota	Pseudomonadota	Verrucomicrobiota	Bacteria diversity	Ref.
Maternal obesity		*Bifidobacterium* spp*.* ↓	*Bacteroide*s spp. ↑	*Staphylococcus* spp. ↑ Lachnospiraceae ↑	Enterobacteriaceae ↑		↓	(8, 11)
Gestational Age	Pre-term birth	*Bifidobacterium* spp*.* ↓	*Bacteroides* spp. ↓	*Staphylococcus* spp. ↑	Enterobacteriaceae ↑ *Enterococcus* spp*.* ↑		↓	(8, 11, 12)
	Full-term birth	*Bifidobacterium* spp. ↑	*Bacteroide*s spp. ↑				↑	(12)
Type of delivery	Vaginal delivery	*Bifidobacterium* spp. ↑	*Prevotella* spp. ↑ *Bacteroides fragilis* ↑	*Lactobacillus* spp. ↑ *Streptococcus* spp. ↑	*Escherichia* spp. ↑		↑	(8, 11–13)
	C-section	*Bifidobacterium* spp*.* ↓ *Propionibacterium* spp. ↑ *Corynebacterium* spp. ↑		Clostridium I ↑ *Clostridium difficile* ↑ *Staphylococcus* spp. ↑	Enterobacteriaceae ↑		↓	(8, 11–13)
Type of feeding	Breastfeeding	*Bifidobacterium* spp. ↑		*Lactobacillus* spp. ↑			↑	(11, 12)
	Formula feeding	*Atopobium* spp. ↑	*Bacteroide*s spp. ↑	*Staphylococcus* spp. ↑	Enterobacteriaceae ↑		↓	(11, 12)
Weaning		*Bifidobacterium* spp. ↓	*Bacteroide*s spp. ↑	Lachnospiraceae ↑ Oscillospiraceae ↑ *Lactobacillus* spp. ↓	Enterobacteriaceae ↓	*Akkermansia* spp. ↑	↑	(11)
Older siblings		*Bifidobacterium* spp. ↑					↑	(11)
Antibiotic treatment	Macrolide		*Bacteroide*s spp. ↑	↓	Enterobacteriaceae ↑		↓	(14)
	Vancomycin			*Lactobacillus* spp. ↓ *Clostridium* spp. ↓			↓	(14)
	Ciprofloxacin			*Faecalibacterium* spp. ↓ Oscillospiraceae ↓			↓	(14)
	Clindamycin						↓	(14)
	Ampicillin		*Bacteroides* spp. ↓	Clostridium I ↑ *Streptococcus* spp. ↓	*Enterococcus* spp*.* ↑		↓	(14)
PPIs treatment		*Bifidobacterium* spp. ↓		*Lactobacillus* spp. ↓ *Clostridium difficile* ↑	Enterobacteriaceae ↑ *Haemophilus* spp. ↑			(8, 15)
Smoking exposure				Ruminococcus spp. ↑		*Akkermansia* spp. ↑		(8)
Diet	High-fat		*Bacteroide*s spp. ↑	Lachnospiraceae ↓ Oscillospiraceae ↓	Enterobacteriaceae ↑ *Enterococcus* spp*.* ↑			(16, 17)
	Animal-based proteins	*Bifidobacterium* spp. ↓	*Bacteroide*s spp. ↑ *Alistipes* spp. ↑	Lachnospiraceae ↓ Oscillospiraceae ↓	Enterobacteriaceae ↑ *Enterococcus* spp*.* ↑			(11, 16, 17)
	Plant proteins			*Lactobacillaceae* ↑				(17)
	Complex carbohydrates		*Prevotella* spp. ↑	Lachnospiraceae ↑ Oscillospiraceae ↑				(16, 17)
	Food additives		*Bacteroides* spp. ↑ Porphyromonadaceae ↑	*Lactobacillus* spp. ↓	Enterobacteriaceae ↑ Enterococcus spp. ↑			(17)

Gut microbiome is physically and functionally linked to the immune system; indeed, some beneficial bacteria that produce immunomodulatory compounds, such as the short-chain fatty acid (SCFA) butyrate, increase the rate of regulatory T cells (Tregs) (i.e., crucial cells involved in immune tolerance) (18). By contrast, the loss of beneficial bacterial species has been associated not only with a reduction in these beneficial compounds but also the production of pro-inflammatory mediators, such as lipopolysaccharides (LPSs), that can contribute to the development of FA (19).

All these data suggest that the balance of beneficial and detrimental bacteria in the GM could be involved in the occurrence of FA, which paves the way to innovative preventive and therapeutic strategies for one of the most prevalent and expensive diseases of the pediatric age (20). This scoping review analyzes the current evidence on GM features associated with pediatric FA and highlights the importance of the GM as a potential target of intervention for preventing and treating this common condition in the pediatric age.

## 2 Methods

### 2.1 Search strategy

The Embase and PubMed databases were searched with medical subject heading (MeSH) keywords to identify studies of interest. The search was last performed on 1 March 2024. The following MeSH terms were used: (gut microbiome OR dysbiosis OR gut microbiota OR microbiome signatures) AND (food allergy OR IgE-mediated food allergy OR food protein-induced allergic proctocolitis OR food protein-induced enterocolitis OR non-IgE food allergy OR cow milk allergy OR hen egg allergy OR peanut allergy OR fish allergy OR shellfish OR tree nuts OR soy allergy OR wheat allergy OR rice allergy OR food sensitization).

The filters used for all databases were language (English only) and time (articles retrieved between 1 January 2011 and 29 February 2024). In addition, the filters applied for PubMed were studies on humans and age (child, birth–18 years; newborn, birth–1 month; infant, birth–23 months; infant, 1–23 months; child, 6–12 years; adolescent, 13–18 years; and preschool child, 2–5 years). Additional filters used for Embase were studies on humans and age (newborn, infant, child, preschool child, school child, and adolescent).

**Figure 1:**
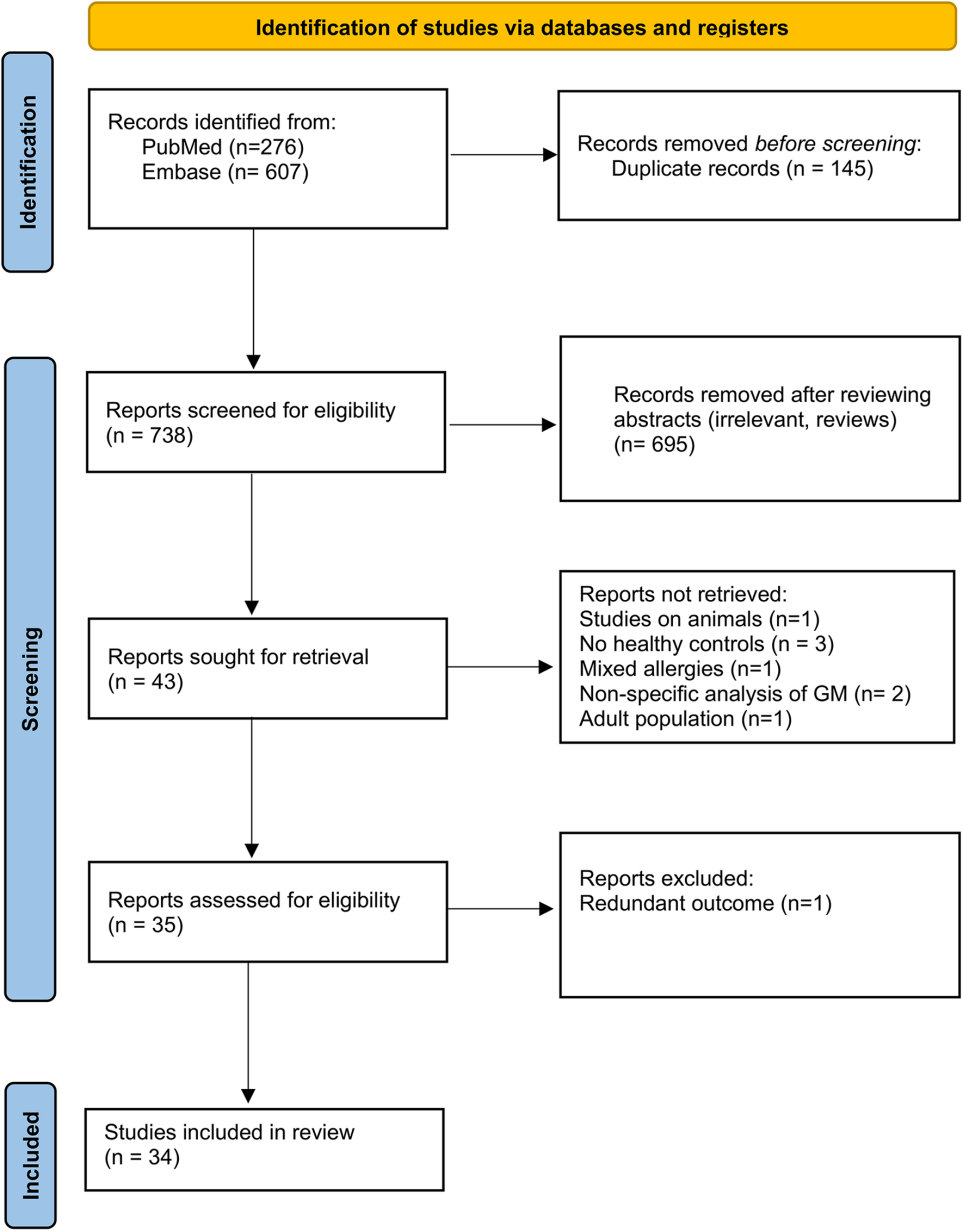
Flow diagram of the identified studies.

This study was conducted following the recommendations of the Preferred Reporting Items for Systematic Reviews and Meta-Analyses (PRISMA) guidelines (21). The search strategy and PRISMA flow chart are detailed in Figure 1.

### 2.2 Selection criteria and data extraction

Two investigators (MF and LC) independently reviewed the titles and abstracts of the original articles extracted in the initial search and selected the observational studies, clinical trials, cross-sectional studies, and case–control studies regarding GM composition and its alterations in pediatric FA and food sensitization in comparison with age- and sex-matched healthy controls that were available as full-text original peer-reviewed articles. Reviews were excluded. In the included studies, the analysis was performed on the stool microbiome. Only pediatric subjects (aged 0–18 years), irrespective of gender, race, geographic location, mode of delivery, and feeding, were chosen for this review. All types of pediatric FA were included regardless of the allergen causing the disease and were as follows: IgE- (including food sensitization) and non-IgE-mediated FA. The main technique used for analyzing GM composition in the included studies was 16S rRNA sequencing or shotgun sequencing due to the comprehensive profiling of the microbiome that these methods allow. The relevant data from the included studies were extracted independently by MF and LC. Any disagreements were solved by discussion, and if not resolved, by consulting a third member of the team (RC).

The bacteria were classified into phylum, family, genus, and species. If the study only offered one of the taxa classifications (i.e., the genus), the National Center for Biotechnology Information (NCBI) taxonomy browser for bacteria was used to determine the rest of the categories upstream of the one specified. NCBI's up-to-date taxonomic classification was used, and this resulted in some phyla and genera being renamed and reclassified.

### 2.3 Data items

The data extracted included the number of participants in the FA and healthy groups, age of subjects, geographical location, methodology of microbiome analysis used, study design, FA features, and GM signature.

## 3 Results

### 3.1 Studies record

As reported in Figure 1, the search yielded a cumulative total of 883 records. After duplicate removal, in the screening phase, 34 papers were included. Studies on animals, studies that did not include a healthy control population, and studies describing participants as “allergic” without specifying the type of allergy were excluded. However, studies involving animals with the primary objective of transferring human stools into gnotobiotic mice were included if microbiome analysis was conducted before the transfer. Studies using supplemented feeding formulas to assess their effect on microbiome composition were excluded unless baseline microbiome analysis was also performed prior to treatment. The characteristics of the studies identified as eligible evidence for this scoping review are presented in Table 2.

**Table 2:** The main characteristics of the studies included in the scoping review.

Study population									
Year	Author (reference)	Design	Type of food allergy	Age (months)	Allergic (*n*)	Controls (*n*)	Method of microbiome analysis	Allergens	Geographical location
2014	Ling et al. (22)	Case–control	IgE, non-IgE	5^a^	34	45	16S rRNA V1-V3 regions pyrosequencing	Mix	China
2018	Dong et al. (23)	Prospective cohort	IgE, non-IgE	2.9^a^	60	60	16S rRNA V3-V4 regions pyrosequencing	CMP	China
2021	Łoś-Rycharska et al. (24)	Case–control	IgE, non-IgE	3.4^a^	54	28	16S rRNA V3-V4 regions Illumina	CMP	Poland
2022	Wang et al. (25)	Prospective cohort	IgE, non-IgE	3 and 6; 1 and 3 days	24	44	16S rRNA V3-V4 regions Illumina	CMP	Norway
2023	Yu et al. (26)	Case–control	IgE, Non-IgE	<6	25	25	16S rRNA V3-V4 regions Illumina	CMP	China
2023	Yan et al. (27)	Case–control	IgE, Non-IgE	14.5^a^	10	10	16S rRNA V3-V4 regions Illumina	Mix	China
2019	Mauras et al. (28)	Case–control	IgE, Non-IgE	9.8^a^	5	6	16S rRNA sequencing	CMP	United Kingdom
2015	Azad et al. (29)	Prospective cohort	IgE	3 and 12	12	154	16S rRNA V4 region Illumina	Mix	Canada
2022	Zhang et al. (30)	Case–control	IgE	102	51	8	16S rRNA V4 region Illumina	Peanut	USA
2017	Tanaka et al. (31)	Prospective cohort	IgE	1, 2, 6 and 12	14	27	16S rRNA V1-V2, V6-V8 regions Illumina and pyrosequencing	Mix	Japan
2018	Kourush et al. (32)	Case–control	IgE	>12	22	20	16S rRNA V4 region, Illumina	Mix	USA
2021	De Fillipis et al. (33)	Case–control	IgE	58^a^	55	29	shotgun sequencing	Mix	Italy
2020	Goldberg et al. (34)	Case–control	IgE	77^b^	233	58	16S rRNA V4 region, Illumina	Mix	Israel
2016	Berni Canani et al. (35)	Clinical study	IgE	4.2^a^	19	20	16S rRNA V4 region, Illumina	CMP	Italy
2015	Chen et al. (36)	Case–control	IgE	13.6^a^	23	22	16S rRNA V3-V5 regions pyrosequencing	Mix	Taiwan
2018	Fazlollahi et al. (37)	Case–control	IgE	9.7^a^	66	75	16S rRNA V4 region, Illumina	Egg	USA
2018	Savage at al. (38)	Case–control	IgE	3 and 6	85	131	16S rRNA V3-V5 regions Roche 454	Mix	USA
2019	Abdel Gadir et al. (39)	Case–control	IgE	1–6, 7–12, 13–18, 19–24, 25–30	56	98	16S rRNA V4 region, Illumina	Mix	USA
2021	Mennini et al. (40)	Case–control	IgE	13^a^	14	14	16S rRNA V3-V4 regions, Illumina	CMP	Italy
2021	Lee et al. (41)	Case–control	IgE	70.8^a^	33	27	16S rRNA V3-V4 regions, Ion S5 XL	Mix	Australia
2017	Inoue et al. (42)	Case–control	IgE	48^a^	4	4	16S rRNA V3-V4 regions, Illumina	Mix	Japan
2011	Chagoyan et al. (43)	Case–control	IgE	7^a^	46	46	FISH and flow cytometry using 16S rRNA probe hybridization and specific bacterial group probes	CMP	Spain
2021	Joseph et al. (44)	Prospective cohort	IgE	1 and 6	44	403	16S rRNA V4 region Illumina	Mix	USA
2016	Guo et al. (45)	Case–control	IgE	78^a^	12	12	PCR-DGGE using universal and species specific primers	CMP	China
2019	Yamagishi et al. (46)	Case–control	IgE	42^b^	18	22	16S rRNA sequencing	Egg	Japan
2018	Dong et al. (47)	Case–control	IgE	76.8^a^	6	8	16S rRNA V3-V4 regions, Illumina	CMP	China
2022	Martin et al. (48)	Prospective cohort	Non-IgE	1, 2, 4, 6 and 12	81	79	16S rRNA V4 region, Illumina	CMP	USA
2018	Berni Canani et al. (49)	Clinical study	Non-IgE	11.4^a^	23	23	16S rRNA V3-V4 regions, Illumina	CMP	Italy
2023	Su et al. (50)	Prospective cohort	Non-IgE	0–3, 4–6, 7–9 and 10–12	8	77	16S rRNA V4 region sequencing	Mix	USA
2018	Díaz et al. (51)	Prospective cohort	Non-IgE	17^a^	17	10	16S rRNA sequencing	CMP	Spain
2022	Caparrós et al. (52)	Case–control	Non-IgE	86.4^a^	17	12	16S rRNA sequencing	Mix	Spain
2012	Kumagai et al. (53)	Case–control	Non-IgE	3	15	15	T-RFLP and qPCR using species specific primers	CMP	Japan
2021	Wang et al. (54)	Case–control	Non-IgE	3.6^a^	3	3	16S rRNA V3-V4 regions, Illumina	CMP	China
2020	Aparicio et al. (55)	Case–control	Non-IgE	7^a^	10	8	16S rRNA V3-V4 regions, Illumina	CMP	Spain

^a^ Mean.

^b^ Median.

### 3.2 Study characteristics

Of the 34 studies included in the review, most (*n* = 24) were case–control studies, eight were prospective cohort studies and two were randomized controlled trials. The year of publication ranged from 2011 to 2023. In terms of geographical region, eight were conducted in the USA, seven in China, four in Italy, four in Spain, four in Japan, and the others were conducted in Poland, Norway, the United Kingdom, Canada, Israel, Taiwan, and Australia. The sample size of the studies ranged from 6 to 447 subjects, representing a total population of 2,822 study participants.

Seven studies enrolled patients with both IgE-mediated and non-IgE-mediated FA without distinguishing between these two categories. Nineteen studies focused on patients with only IgE-mediated FA or food sensitization, and eight studies enrolled patients with non-IgE-mediated FA.

## 4 Gut microbiome structure

All studies included in our review reported alterations in the structure of the GM in pediatric FA patients and in subjects sensitized to food allergens compared with healthy controls. No differences were reported according to the sex of the subjects and the food antigens responsible for the disease. Most of the available data referred to patients already affected by FA; only five studies prospectively investigated the structure of the GM and the subsequent occurrence of FA (25–50). These studies showed that GM dysbiosis could precede the occurrence of FA by 3–6 months and persist during the disease course (25–50). Among these studies, Martin et al. (48) analyzed a population of children affected by food protein-induced allergic proctocolitis (FPIAP) and compared GM features before (pre-symptomatic), during (symptomatic), and after the resolution of the disease. Interestingly, they reported a progressive increase in the abundance of the Enterobacteriaceae family from the pre-symptomatic group to the symptomatic stage (48). Moreover, in the same study, an increased abundance of the *Lactobacillus* genus was observed when comparing children with active disease with children who acquired immune tolerance, supporting the hypothesis that these bacteria could be linked to the development of immune tolerance (48). Joseph et al. prospectively evaluated the structure of the GM in children who developed IgE-mediated FA or who did not develop FA (healthy controls), collecting stool samples at 1 and 6 months of life. The results of the study highlighted an overall delayed maturity of the structure of the GM as a typical feature of the intestinal microbial community in FA children (44).

Four studies investigated the structure of the GM in children with food antigen sensitization (29, 36, 38, 40). In food-sensitized children, there is a lower overall GM richness at 3 months of life and an increased Enterobacteriaceae/Bacteroidaceae ratio (E/B ratio), before the development of sensitization at 12 months (29). Accordingly, Chen et al. found that food-sensitized subjects exhibited a lower total microbiota diversity (*p* = 0.01) and a lower diversity of the bacterial phylum Bacteroidetes (*p* = 0.02) compared with healthy controls (36). On the contrary, no differences in microbial diversity measures were detected between food sensitization and food allergy cases and controls. However, at the genus level, *Haemophilus*, *Dialister*, *Dorea*, and *Clostridium* were significantly underrepresented among subjects with food sensitization (40). Finally, in a prospective study comparing cow's milk-sensitized subjects vs. cow's milk allergy patients vs. healthy controls, sensitized subjects had the highest number of total and rare operational taxonomic units (OTUs) (i.e., observed and Chao I index, respectively), whereas cow’s milk allergy was characterized by the lowest OTU richness (38).

Considering the relevant role elicited by the age of the subject on the maturation of the GM (11, 56), we divided the included studies according to the age of the enrolled subjects. We classified the available data into the following three categories according to the participant's age: (1) from 0 to 6 months; (2) from 6 to 12 months; and (3) >12 months of age. All data obtained in our study are summarized in Tables 3–8, which show the bacterial taxa that were found to be increased or reduced in at least two studies.

**Table 3:** Bacterial species overrepresented in the GM of children affected by FA in the first 6 months of life.

Overrepresented in FA: 0–6 months			
Phylum	Family	Genus	Species
Bacteroidota (24, 25, 50)	Tannerellaceae	*Parabacteroides* (24)	*distasonis* (50)
	Bacteroidaceae	*Bacteroides* (24)	*fragilis* (50), *caccae* (50), *ovatus* (50)
	Dysgonomonadaceae	*Dysgonomonas* (26, 54)	
Bacillota	Clostridiaceae (22, 26, 54)	*Clostridium* (22, 26, 39, 54)	*baratii* (50), *perfringens* (50)
	Lactobacillaceae	*Lactobacillus* (22, 39)	
	Oscillospiraceae (22, 35)	*Faecalibacterium* (22, 35)	
		*Flavonifractor* (22, 39)	
	Turicibacteraceae	*Turicibacter* (25, 35)	
Pseudomonadota (22, 23, 26, 29)	Enterobacteriaceae (23, 29, 31, 48, 54)	*Escherichia* (22, 24, 25, 48, 54)	*coli* (25)
		*Klebsiella* (26, 53, 54)	
		*Enterobacter* (25, 31, 54)	
	Xanthomonadaceae (26)	*Stenotrophomonas* (38)	
Actinomycetota (22, 26)	Bifidobacteriaceae	*Bifidobacterium* (22, 54)	
	Eggerthellaceae	*Rhodococcus* (22, 54)	
Verrucomicrobia (37)	Akkermansiaceae	*Akkermansia* (24)	

The studies follow the numbering used in Table 2.

**Table 4:** Bacterial species that are underrepresented in the GM of children affected by FA in the first 6 months of life.

Underrepresented in FA: 0–6 months			
Phylum	Family	Genus	Species
Bacteroidota (26)	Bacteroidaceae (26, 29, 44)	*Bacteroides* (22, 23, 26, 29)	*fragilis* (53)
Bacillota (26, 50)	Clostridiaceae (48)	*Clostridium* (24, 38, 48)	*neonatale* (50), *butyricum* (50)
	Oscillospiraceae (23, 26, 48)	*Clostridium*	*leptum* (53)
		*Faecalibacterium* (25, 36)	*prausnitzii* (50)
	Lactobacillaceae (31, 54)	*Lactobacillus* (31, 54)	
	Streptococcaceae	*Lactococcus* (24, 25, 38)	*lactis* (47)
		*Streptococcus* (22, 24, 25, 35)	
	Veillonellaceae (26)	*Veillonella* (22, 31, 50)	
	Lachnospiraceae (22, 25, 26, 39, 54)	*Lachnospiraceae* (22)	
		*Dorea* (26, 38)	
		*Blautia* (22, 26)	*coccoides* (52)
		*Fusicatenibacter*	*saccharivoran*s (24)
		*Ruminococcus* (25, 54)	*gnavus* (50)
		*Roseburia* (25)	*faecis* (50)
	Selenomonadales (26)	*Megamonas* (22)	
	Enterobacteriaceae (35)	*Enterococcus* (31)	
	Staphylococcaceae	*Staphylococcus* (35)	
	Erysipelotrichaceae (22, 25, 26)	*Holdemania* (25)	
Pseudomonadota (22)	Alcaligenaceae (22, 54)	*Alcaligenes* (25)	
	Xanthomonadaceae (22, 54)	*Stenotrophomonas* (25)	
	Enterobacteriaceae (26)	*Klebsiella* (22, 54)	
		*Escherichia* (35, 53)	*coli* (53)
	Pasteurellaceae (26)	*Haemophilus* (24, 26, 38)	
Actinomycetota (23, 25, 50)	Bifidobacteriaceae (23, 25, 38, 44)	*Bifidobacterium* (24, 25, 35, 50)	*adolescentis* (50)
	Micrococcaceae (35)	*Rothia*	*mucilaginosa* (50)

The studies follow the numbering used in Table 3.

**Table 5:** Bacterial species overrepresented in the GM of children affected by FA from 6 to 12 months of life.

Overrepresented in FA: 6–12 months			
Phylum	Family	Genus	Species
Bacteroidota (49, 50)	Tannerellaceae	*Parabacteroides*	*distasonis* (50)
	Bacteroidaceae	*Bacteroides* (28, 50)	*fragilis* (50), *Ovatus* (50), *caccae* (50), *dorei* (28), *thetaiotaomicron* (28), *ovatus* (28), *uniformis* (28), *vulgatus* (28)
Bacillota (37)	Lachnospiraceae (5, 35)	*Blautia* (49)	*coccoides* (43)
		*Faecalibacterium* (37, 39)	
	Clostridiaceae	*Clostridium* (31)	*baratii* (50), *perfringens* (50), *paraputrificum* (31), *tertium* (31)
	Peptostreptococcaceae (55)	*Romboutsia* (39)	
Pseudomonadota (29)	Enterobacteriaceae (29, 31, 48)	*Escherichia* (31, 48)	

The studies follow the numbering used in Table 3.

**Table 6:** Bacterial species that are underrepresented in the GM of children affected by FA from 6 to 12 months of life.

Underrepresented in FA: 6–12 months			
Phylum	Family	Genus	Species
Bacteroidota	Bacteroidaceae (29)	*Bacteroides* (29, 48, 55)	
Bacillota (50)	Clostridiaceae	*Clostridium* (28, 48)	*neonatale* (50), *perfringens* (28), *butyricum* (28, 50)
	Oscillospiraceae (48)	*Faecalibacterium*	*prausnitzii* (50)
	Lactobacillaceae (37)	*Lactobacillus* (28)	
Actinomycetota (50)	Bifidobacteriaceae (43, 55)	*Bifidobacterium* (28, 39, 50, 55)	*adolescentis* (50)

The studies follow the numbering used in Table 3.

**Table 7:** Bacterial species overrepresented in the GM of children affected by FA with more than 12 months of life.

Overrepresented in FA: >12 months			
Phylum	Family	Genus	Species
Bacteroidota (45, 47)	Tannerellaceae	*Parabacteroides* (32, 33, 41)	
	Bacteroidaceae	*Bacteroides* (39, 41, 47)	*thetaiotaomicron* (30)
	Prevotellaceae	*Prevotella* (36–38)	
	Rikenellaceae	*Alistipes* (32, 41)	
Bacillota (36, 47)	Lachnospiraceae (34, 41, 52)	*Blautia* (33, 34, 52)	*wexlerae* (33), *obeum* (34), *coccoides* (45)
		*Ruminococcus* (33, 36, 39)	*gnavus* (33)
		*Anaerobutyricum*	*hallii* (36, 52)
	Clostridiaceae	*Clostridium* (36, 45)	
	Oscillospiraceae (34, 36, 40, 41, 47)	*Faecalibacterium* (33, 34, 47)	*prausnitzii* (33)
		*Oscillibacter* (32, 34)	*valericigenes* (34)
		*Subdoligranulum* (33, 36, 47)	
	Lactobacillaceae	*Lactobacillus* (36)	*fermentum* (45)
	Streptococcaceae	*Streptococcus* (40, 41)	
Pseudomonadota (36)	Enterobacteriaceae (46)	*Escherichia* (27, 36)	*coli* (45)
	Sphingomonadaceae	*Sphingomonas* (36, 40)	
	Sutterellaceae	*Parasutterella* (36, 45)	
Actinomycetota	Coriobacteriaceae	*Collinsella* (34, 36)	*aerofaciens* (34)
Verrucomicrobia	Akkermansiaceae	*Akkermansia* (27, 36, 40)	

The studies follow the numbering used in Table 3.

**Table 8:** Bacterial species that are underrepresented in the GM of children affected be FA with more than 12 months of life.

Underrepresented in FA: >12 months			
Phylum	Family	Genus	Species
Bacteroidota (22, 23, 28, 51)	Bacteroidaceae (51, 52)	*Bacteroides* (34, 36, 51)	*vulgatus* (33), *ovatus* (52)
	Prevotellaceae	*Prevotella* (34, 36)	*copri* (34)
	Rikenellaceae (32)	*Alistipes* (36, 39, 41)	
	Tannerellaceae	*Parabacteroides* (36, 40)	
Bacillota	Lachnospiraceae (41)	*Dorea* (39, 42)	
Actinomycetota (32, 47, 51)	Bifidobacteriaceae (45, 51)	*Bifidobacterium* (27, 45, 47, 51)	*adolescentis* (34), *longum* (33), *catenulatum* (45)
Thermodesulfobacteriota	Desulfovibrionaceae	*Bilophila* (39, 45)	

The studies follow the numbering used in Table 3.

At the phylum level, we found that in children affected by FA aged 0–6 months there was an increase in Pseudomonadota (22, 23, 26, 29) and a decrease in Actinomycetota (23, 25, 50). In children aged >12 months there was a decrease in the phylum Bacteroidota (22, 23, 40, 51).

At the family level, we found an increase in Enterobacteriaceae within the GM of FA children (22–25, 29, 30, 36, 45, 48, 49, 53, 54). In particular, the species *Escherichia coli* was overrepresented. The Enterobacteriaceae family was one of the most represented taxa in the GM of the healthy subjects in the first 6 months of life and its abundance gradually decreased with growth. By contrast, this shift was not reported in FA children, resulting in an increase in the Enterobacteriaceae/Bacteroides ratio (23, 29), resembling the features of an immature microbial ecosystem (57).

During the first 6 months of life, we also noticed an increase in the Clostridiaceae family with some species that were overrepresented, such as *Clostridium baratii*, *Clostridium perfringens*, *Clostridium paraputrificum*, and *Clostridium tertium* (22, 31, 39, 54), and other species that were decreased, such as *Clostridium neonatale* and *Clostridium butyricum*, in FA children compared with healthy controls (28, 31, 50). A decrease in Streptococcaceae (the genera *Lactococcus* and *Streptococcus*) (22, 24, 25, 31, 35, 36, 38, 55) and Erysipelotrichaceae (22, 25, 26) was also found in FA children aged 0–6 months.

Another common FA feature was the reduction in the beneficial Bifidobacteriaceae family reported in all ages (23, 25, 28, 33–35, 38, 39, 44, 45, 47, 50, 51, 55). In particular, the *Bifidobacterium* genus, which belongs to this family, is an important source of beneficial immunomodulatory compounds, such as SCFAs, and constitutes one of the prevalent taxa in the first months of life in healthy breastfed infants (11).

Moreover, we found a clear reduction in the *Lactobacillaceae* family (the genera *Lactobacillus* and *Weissella*) in the GM of FA children, especially in the first 6 months of life (24, 28, 31, 54). The decrease in this family could precede the onset of FA. In addition, in children affected by cow milk protein allergy (CMPA), it has been demonstrated that the total abundance of the *Lactobacillaceae* family increases with the development of immune tolerance (25).

The Bacteroidaceae family was decreased in the GM of FA children (26, 29, 44, 51, 52) but there are conflicting data on the genus *Bacteroides*. This could be related to the large number of species belonging to this genus. According to some studies, the species *Bacteroides thetaiotaomicron*, *Bacteroides uniformis*, *Bacteroides ovatus*, *Bacteroides caccae*, and *Bacteroides fragilis* are overrepresented in the GM of FA children (22, 24, 28, 30, 39, 41, 49, 50). In other studies, other species belonging to the *Bacteriodes* genus were underrepresented in FA children (23, 29, 33, 37, 44, 48, 51, 53–55), e.g., *Bacteroides vulgatus*, *Bacteroides dorei*, and *B. fragilis*. In addition in this case, some species (e.g., *B. fragilis*) were overrepresented (50) or underrepresented (53) in FA children, complicating the interpretation of this aspect. These apparently conflicting data could be explained by the strain-specificity that was not considered in these studies (58).

Lachnospiraceae is another pivotal family that can distinguish FA vs. healthy children. In particular, the genera *Lachnospira* (33, 42), *Anaerostipes* (22, 33, 42), *Lachnobacterium* (50), and *Ruminococcus* (33, 36, 39) were increased in the microbial ecosystem of the FA children aged more than 6 months (28, 34, 41, 52, 55), whereas this family was decreased in the first 6 months of life (22, 25, 26, 39, 54). *Dorea* and *Blautia*, two bacterial genera belonging to this family, were decreased in FA children compared with healthy controls in the first 6 months of life (22, 26, 38, 52). According to some studies, the increase in some members of the Lachnospiraceae family could precede the development of immune tolerance, as also demonstrated in children with CMPA (35). Conversely, in another study, the relative abundance at baseline of Lachnospiraceae was significantly higher in infants who did not outgrow CMPA than in infants who outgrew CMPA (59).

Regarding the Oscillospiraceae family, there is still contrasting evidence and no agreement in the selected studies. In FA pediatric patients, this family was underrepresented in the first 6 months of life (23, 26, 48) and overrepresented in children aged >6 months (22, 35, 36, 40).

One of the main species involved in SCFA metabolism, *Faecalibacterium prausnitzii*, has been found to be both increased (33, 34, 37, 39, 47) and decreased (26, 38, 50) in FA children in relation to age. The metagenomic approach once again clarified that the same bacterial species can exert different actions. In a previous study by our group, we observed the abundance of this bacterium in the GM of FA children, and we also described the presence of different clades of *F. prausnitzii* in healthy and allergic children (clade A being characteristic of allergic children), characterized by distinct metabolic activities (33). Conversely, according to the two studies included in the review that analyzed the GM composition in children affected by food protein-induced enterocolitis syndrome (FPIES), the abundance of *F. prausnitzii* was decreased (50, 52). In pediatric FA patients aged >12 months, two other bacterial genera, *Akkermansia* (32, 34, 42) and *Bilophila* (39, 45), were decreased.

## 5 Discussion

In this scoping review, we provided updated information on GM features associated with the occurrence of pediatric FA. In addition, we provided data on the structure and function of the GM at different ages and information regarding the most relevant environmental factors impacting them. The final aim was to make data accessible and engaging for physicians and researchers to help the development of innovative, preventive, and therapeutic strategies for managing FA.

Our results confirmed the presence of alterations in the structure and function of the GM in pediatric patients affected by FA. Impaired GM maturation, marked by a reduction in age-related GM maturation, is a hallmark of FA occurrence (60, 61). FA children have a lower GM diversity, and their microbial ecosystem is unbalanced, with a loss of beneficial species and an increase in species associated with alterations in the gut barrier, inflammation, and the Th2 response (61). Data regarding selected bacteria species seem to be conflicting but these discrepancies should be interpreted considering that bacteria belonging to the same species can exert both beneficial and detrimental effects on the gut barrier and immune system (33, 58, 62).

The term “dysbiosis” refers to the disruption of the normal homeostasis of the microbial ecosystem inhabiting the human intestinal tract, involving quantitative and qualitative alterations in the predominant bacterial species (63). Dysbiosis, however, does not merely mean alterations in the structure but also in GM function, which stimulates the immune system in a pro-inflammatory direction (64). This condition precedes the occurrence of FA (48) and is driven by the influence of several environmental factors (see Table 1).

**Figure 2:**
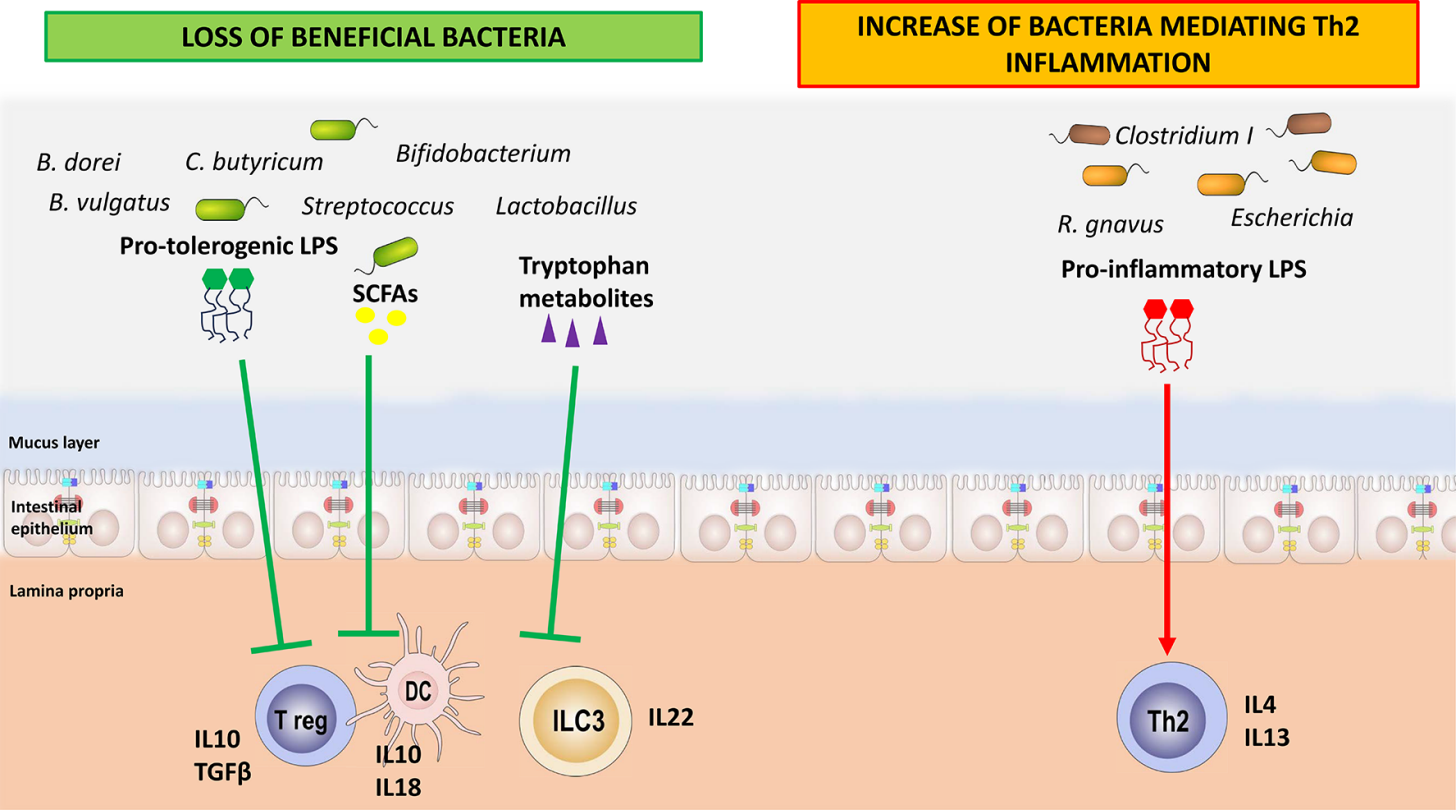
How the loss of beneficial bacteria and the increase in harmful bacteria may play a role in food allergy pathogenesis. The loss of beneficial bacteria and of their immune-regulatory metabolites leads to a breakdown in immune tolerance mechanisms. Concomitantly, the higher rate of harmful bacteria is associated with a huge release of pro-inflammatory compounds, such as specific lipopolysaccharides, resulting in the activation of T helper 2 cells.

Several mechanisms of action have been proposed to explain how GM dysbiosis and dysfunction can facilitate the occurrence of FA (see Figure 2). First, the loss of beneficial species, such as *Bifidobacterium*, *Lactobacillus*, and certain species within the Lachnospiraceae family, facilitate the alteration in gut barrier homeostasis. These bacterial strains are responsible for producing SCFAs as butyrate, through the fermentation of non-digestible dietary fibers, which increase gut barrier function by acting on enterocytes and immune cells (65). Metabolomic analyses conducted on stool samples from allergic children demonstrated a reduction in butyrate production (43, 49). The SCFAs exert their effects on the host primarily through G protein-coupled receptors (GPR41, GPR45, and GPR109A) promoting immune tolerance (66). Butyrate serves as an energy substrate for colonocytes, modulating their metabolism and promoting the expression of mucins. By inhibiting histone deacetylases (HDAC), it exerts an epigenetic effect on immune cells such as dendritic cells (DCs), stimulating the production of IL-18, which has a key role in maintaining barrier function, and IL-10 and IL-23. The SCFAs stimulate the proliferation and activation of Treg cells through the same epigenetic mechanism. Among other effects, they facilitate class switching and promote IgA production through B lymphocytes, improving gut barrier function (66). In this way, the reduction of butyrate-producing bacteria has been associated with an imbalance in the Th1/Th2 ratio toward Th2 responses and a decrease in Treg cells, resulting in the loss of oral tolerance and development of FA (67–70).

In our previous study, we described an increase in *Lactobacillus* and SCFA-producing bacteria belonging to the Lachnospiraceae family, such as *Roseburia*, *Coprococcus*, and *Blautia*, in CMPA infants who acquired immune tolerance after a 6-month dietary treatment with extensively hydrolyzed casein formula (EHCF) supplemented with the probiotic *Lactobacillus rhamnosus* GG (35). Martin et al. also described an increased abundance of *Lactobacillus* in children affected by FPIAP who developed immune tolerance (48). In another study, we also found that a higher abundance of Lachnospiraceae (specifically *Lachnospira pectinoschiza* and *Anaerostipes hadrus*) and *Bifidobacterium* in the stools of allergic children at diagnosis correlated with the development of immune tolerance after 36 months of follow-up (33).

Another important class of compounds produced by a healthy GM, identified through metabolomics, are tryptophan metabolites such as indole, indole-3-acetic acid, skatole, indole-3-aldehyde, and indole-lactic acid (67). Several bacterial strains, such as *Lactobacillus* spp., *Bifidobacterium* spp., and *B. fragilis* that are underrepresented in the GM of FA children share this metabolic pathway, which contributes to the maintenance of intestinal homeostasis by preventing colonization by potentially pathogenic organisms and reducing inflammation (66).

Through the activation of the aryl hydrocarbon receptor (AhR), tryptophan derivatives promote the production of IL-22 by type 3 innate lymphoid cells (ILC3s) and ROR*γ*t+ T cells (71). IL-22 belongs to the family of IL-10, the main anti-inflammatory cytokine, and allows the production of antimicrobial peptides and the expression of tight junctions proteins (72). On the other hand, 3-indole-lactic acid inhibits the polarization of CD4+ Th lymphocytes toward the Th17 lineage and instead promotes the development of Treg cells (67).

In addition, the microbiome is responsible for another metabolic activity, less studied in the context of FA, which is the production of secondary bile acids (66). Primary bile acids are produced via host cholesterol catabolism in the liver and are further secreted by the gall bladder into the gut lumen after conjugation (66). Bile acids that escape from reabsorption in the terminal ileum can be metabolized by the GM into secondary bile acids by certain bacterial genera, such as *Lactobacillus*, *Bifidobacterium*, *Bacteroides fragilis*, and some species of *Clostridium* (66). Campbell et al. found that one of these compounds, 3β-hydroxydeoxycholic acid (isoDCA), increased Foxp3 expression in T lymphocytes by acting on DCs in an immunoregulatory way (73). Metabolomic studies on FA and other allergic conditions, such as asthma, have revealed distinct profiles of secondary bile acids, indicating an alteration in this metabolic pathway that is specific for the type of allergic disorder (74).

Gut microbiome interacts with the immune system also through bacterial components such as LPSs and flagellins. Changes to the structure of these bacterial components can have significant impacts on signaling so that they can exert anti-inflammatory or pro-inflammatory activities (75). In particular, LPS is a glycoconjugate part of the outer cell membrane of Gram-negative bacteria (76). Fragments of LPS are released into the environment by the bacteria during the continuous process of outer bacterial membrane renewal (77). Lipid A is the main immunostimulatory portion of LPS (78). It is recognized by the toll-like receptor 4 (TLR4)/myeloid differentiation factor 2 (MD-2) receptor complex, leading to the production of pro-inflammatory mediators through the NF-*κ*B pathway, thus initiating and modulating the immune response. The activation of the TLR4/MD-2-dependent response is largely determined by the chemical structure of lipid A (78). The Enterobacteriaceae family produces an LPS with pronounced pro-inflammatory activity, which can disrupt the gut barrier and drive the production of pro-inflammatory cytokines such as IL-4, IL-13, and TNFα (75). LPS derived from *Bacteroides* sp., which is diminished in stool samples from children affected by FA, does not seem to stimulate this pro-inflammatory pathway. It elicits a tolerogenic response with the increase in IL-10 and TGFβ (79). Capsular polysaccharide A (PSA) of commensal *B. fragilis* can be identified via TLR2 and TLR1 heterodimers together with Dectin, resulting in glycogen synthase kinase 3b (GSK3b) inactivation, which in turn promotes the cAMP response element binding protein (CREB)–associated activation of anti-inflammatory genes and Treg cell differentiation (80).

In a recent study from our group, we purified fecal LPSs and lipid A from healthy children and children affected by IgE-mediated FA (76). We provided evidence for a different immunoregulatory action elicited by LPS on peripheral blood mononuclear cells collected from healthy donors, suggesting that LPSs from healthy individuals can protect against the occurrence of FA, and LPSs from children affected by FA can promote an allergic response (76).

The significance of dysbiosis in the pathogenesis of FA is further reinforced by studies on fecal microbiota transplantation (FMT) (39, 54, 81, 82). FMT of feces from allergic children into germ-free (GF) mice results in a deficit in the total number of Treg lymphocytes in the lamina propria, which are known to play a crucial role in immune tolerance. Specifically, the quantity of Tregs exhibits a positive correlation with the abundance of bacteria from the genera *Bacteroides* and *Lactobacillus*, as well as with the family Lachnospiraceae. Conversely, there is a negative correlation with *Raoultella* and *Clostridium sensu stricto* (54).

Furthermore, the bacterial colonization of GF mice with fecal samples from healthy children shields mice from an anaphylactic response to beta-lactoglobulin challenge, and this response does not occur when fecal samples from CMPA infants are transferred to mice (82). According to this study, the protective action of the healthy microbiome is primarily linked to the abundance of the Lachnospiraceae family (82).

Bacteriotherapy with certain consortia of Clostridiales and Bacteroidales also protects sensitized mice from developing FA symptoms, increasing the number of Tregs in the lamina propria (LP) after FMT (39), thereby supporting the possible protective role of these bacterial strains against FA. Atarashi et al. also demonstrate the ability of some bacterial families belonging to Clostridiales, such as Lachnospiraceae and Oscillospiraceae, to induce Treg cells in a murine model (81).

Finally, to translate the GM concept in clinical practice, it is crucial to define the environmental factors inducing GM dysbiosis and dysfunction, and then the occurrence of FA. In Table 1, we summarize the actual available evidence on environmental factors influencing the alteration in the GM and the occurrence of FA. Since intrauterine life, many environmental factors actively modulate the structure of the microbiota from both the maternal and the fetal side (83). The maternal GM during pregnancy influences the newborn's GM composition, impacting their risk of developing allergic conditions (61). In particular, maternal obesity (8, 11), an unbalanced diet during pregnancy (11, 16, 84), the use of antibiotics during pregnancy (14, 16, 61, 85, 86), and exposure to smoke (8) act from the maternal side, reducing GM biodiversity and increasing detrimental bacteria linked to the occurrence of FA.

Birth is a crucial moment for the colonization of the newborn's gut. Gestational age and the mode of delivery have a huge impact on GM composition (8, 11–13). Pre-term birth and cesarean section (C-section) are linked to the development of allergic conditions (8, 11–13). Vaginally delivered infants meet the maternal vaginal and fecal microbiota, which results in neonatal gut colonization by vagina-associated microbes such as *Lactobacillus* (87). In contrast, C-section-delivered infants are not directly exposed to maternal microbes and are thus more likely to become colonized by environmental microorganisms from the maternal skin, hospital staff, or hospital environment (87). Then, the type of infant feeding (breastfeeding vs. formula feeding) and the diet after weaning play a crucial role in modulating the structure and function of the GM (11, 17, 88).

The housing setting, increased hygiene, and the presence of siblings and pets can also modulate GM composition (11). Broad-spectrum antibiotics can affect the abundances of 30% of the bacteria in the gut community, causing rapid and significant drops in taxonomic richness, diversity, and evenness (89, 90). Antibiotic-induced GM alterations can remain for long periods of time, spanning months and even years (15, 91, 92).

Some therapeutic strategies have been proposed to counteract GM dysbiosis, such as probiotics, prebiotics, postbiotics, and synbiotics (93–96). Several clinical studies and a systematic review and meta-analysis suggest that active diet therapy with EHCF supplemented with the probiotic *L. rhamnosus* GG (LGG) may reduce disease duration in children with CMPA (95–99), the occurrence of functional gastrointestinal disorders (100), and other allergic manifestations (eczema, urticaria, asthma, and rhinoconjunctivitis) later in the life compared with other formulas (95, 96, 98, 101). In addition, substitutive infant formulas have emerged as a major cost driver in the management of pediatric patients with CMPA, as it is estimated to be up to six times more expensive to feed a child with this condition (102, 103). Therefore, these data highlight the role of EHCF + LGG not only in improving clinical outcomes but also in reducing the cost of patient management, providing a cost-effective nutritional strategy for healthcare systems in the management of infants with CMPA (104–106). It is important to underline that the beneficial results observed in FA children treated with LGG could not necessarily be extended to other probiotic strains. Data on other probiotic strains with regard to the modulation of the structure of the GM in FA children are conflicting (107). More evidence is needed to demonstrate the role of these strategies in preventing the onset of FA (108) or alternatively modifying its natural history.

## 6 Conclusion

Our knowledge on the link between GM dysbiosis and FA (i.e., the GM-immune system axis) is continuosly enriched with new insights (109, 110). New investigations are advocated to better define the role of other components of the GM, such as viruses, fungi, and archaea (111, 112), in the regulation of the GM-immune system axis.

It is now clear that GM dysbiosis is strongly associated with immune system dysfunction. Thus, the loss of some beneficial bacteria species in favor of harmful ones is not merely an alteration of the structure of the GM but implies a GM dysfunction that impairs immune system development and activity in the pediatric age. Concomitantly, the finding that several environmental factors actively modulate the GM-immune system axis opens the way for the development of new strategies aimed at preventing and treating FA with a possible positive impact also on the development and treatment of other non-communicable diseases.
