# Corrigendum: Gut microbiome features in pediatric food allergy: a scoping review

**DOI:** 10.3389/falgy.2025.1588779

**Published:** 2025-03-20

**Authors:** Margherita Farnetano, Laura Carucci, Serena Coppola, Franca Oglio, Antonio Masino, Marica Cozzolino, Rita Nocerino, Roberto Berni Canani

**Affiliations:** ^1^ Department of Translational Medical Science, University of Naples Federico II, Naples, Italy; ^2^ ImmunoNutritionLab at the CEINGE Advanced Biotechnologies Research Center, University of Naples Federico II, Naples, Italy; ^3^ Department of Biomedicine and Prevention, University of Rome “Tor Vergata”, Rome, Italy; ^4^ Task Force on Microbiome Studies, University of Naples Federico II, Naples, Italy; ^5^ European Laboratory for the Investigation of Food-Induced Diseases, University of Naples Federico II, Naples, Italy

**Keywords:** microbiota, short-chain fatty acids, probiotics, allergy, dysbiosis, immune tolerance, cow milk protein allergy, children

A Corrigendum on Gut microbiome features in pediatric food allergy: a scoping review By Farnetano M, Carucci L, Coppola S, Oglio F, Masino A, Cozzolino M, Nocerino R and Berni Canani R (2024). Front. Allergy 5. doi: 10.3389/falgy.2024.1438252

Incorrect Funding

In the published article, there was a mistake in the Funding statement section, due to the lack of the CUP codes related to funding. The correction to the Funding Section does not affect the results or conclusions of the article. The authors regret the lack.

The funding statement was displayed as “The authors declare financial support was received for the research, authorship, and/or publication of this article. This study was supported by the Department of Translational Medical Science of the University of Naples “Federico II,” Naples, Italy, which received funding from the National Recovery and Resilience Plan (NRRP), Mission 4 Component 2 Investment 1.3—Call for tender No. 341 of 15 March 2022 of the Italian Ministry of University and Research funded by the European Union—NextGenerationEU; Award Number: Project code PE00000003, Concession Decree No. 1550 of 11 October 2022 adopted by the Italian Ministry of University and Research, CUP D93C22000890001, Project title “ON Foods—Research and innovation network on food and nutrition Sustainability, Safety and Security –Working ON Foods” and from the Italian Ministry of Health–Health Operational Plan Trajectory 5–Line of action “Creation of an action program for the fight against malnutrition in all its forms and for the dissemination of the principles of the diet Mediterranean” [Mediterranean Diet for Human Health Lab (MeDiHealthLab) No. T5-AN-07].”

The correct Funding statement appears below.

